# Supplementary material for: Application of Parallel Reaction Monitoring in 15N Labeled Samples for Quantification
Source: Front Plant Sci. 2022 May 3;13:832585. doi: 10.3389/fpls.2022.832585 (PMC9111532; doi:10.3389/fpls.2022.832585)
Supplement: Supplementary file 1 [file Data_Sheet_1.pdf]

Steps to edit isotope modification:

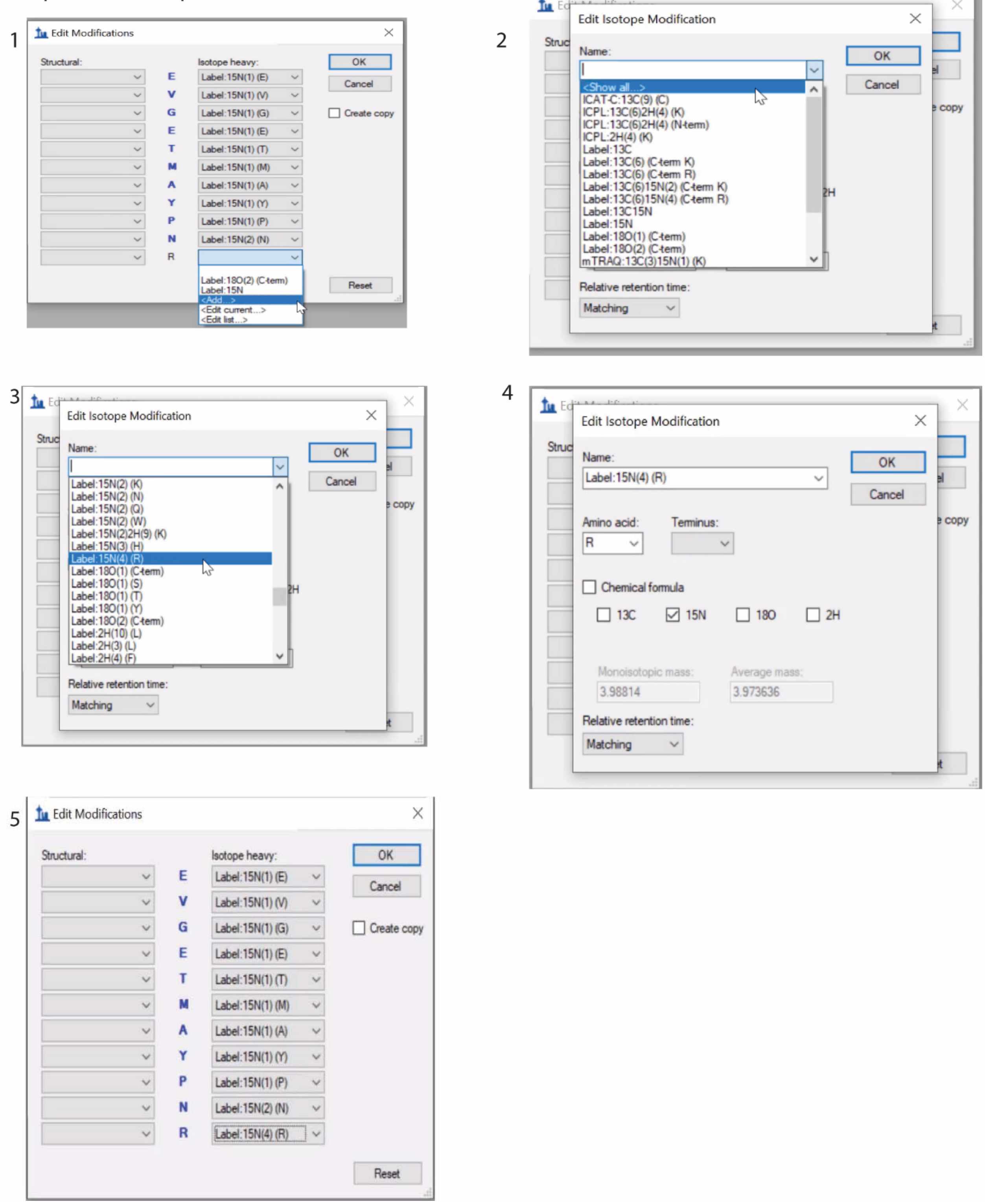

Supplemental Figure 1: Example of steps for adding heavy isotope modification.

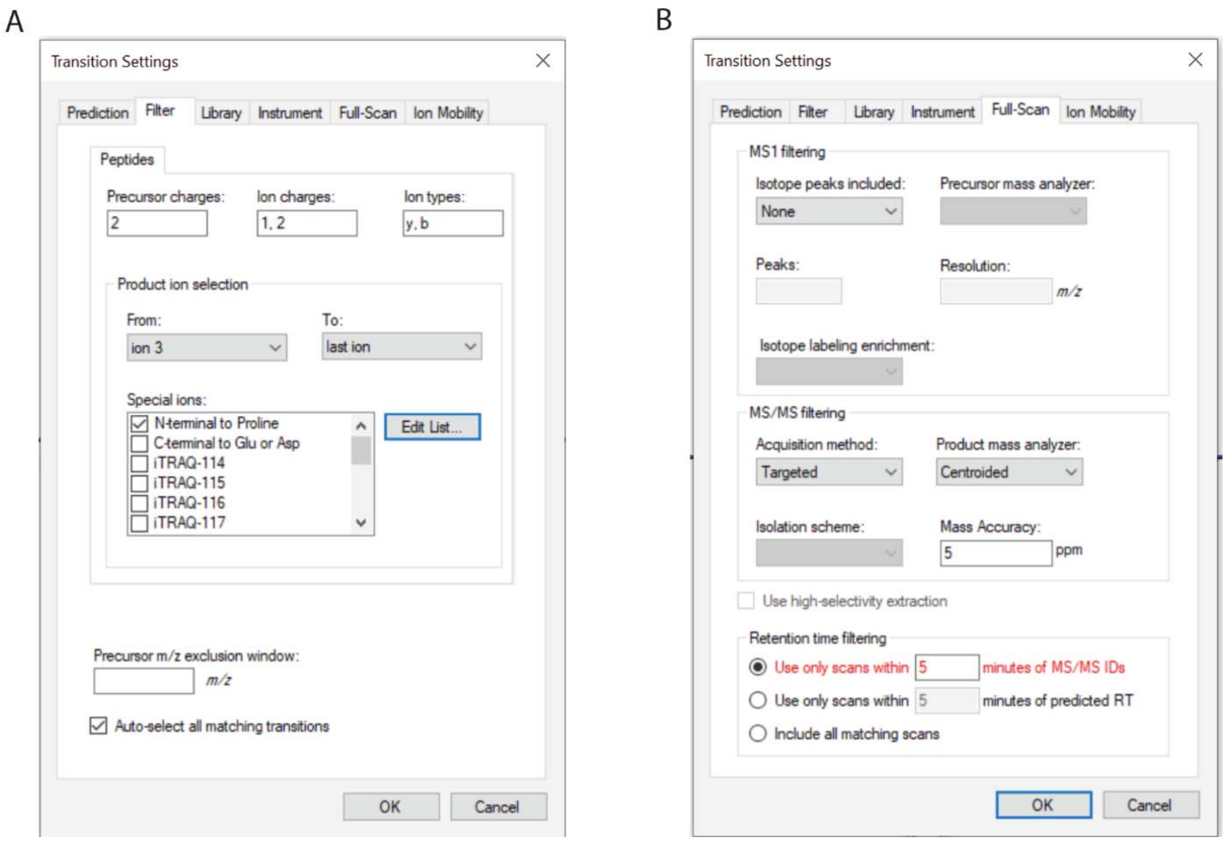

Supplemental Figure 2: Example of setting up transition setting.

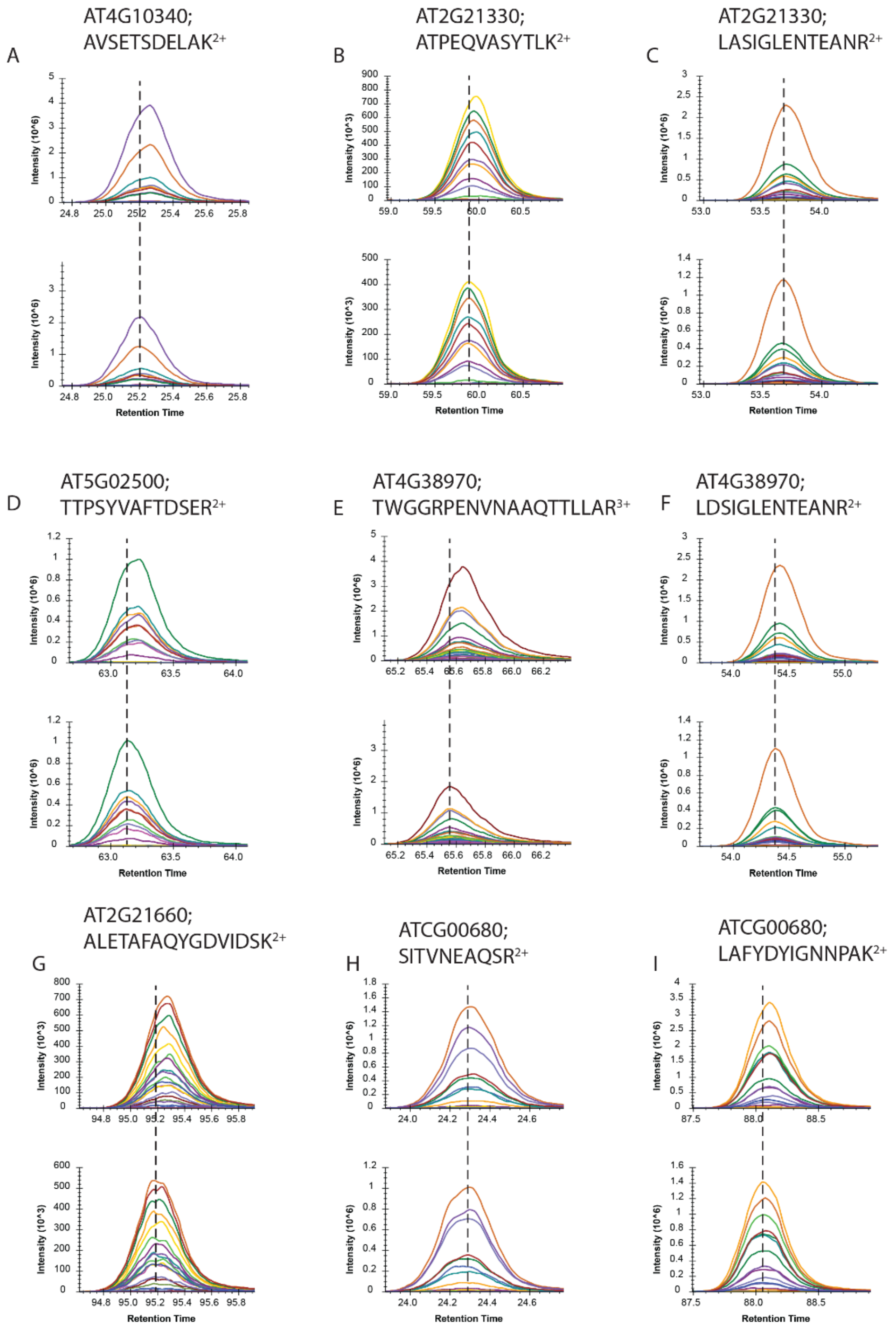

Supplemental Figure 3: PRM quantification on multiple proteins. 42 pairs of peptides (^{14}N and ^{15}N peptides) are quantified using the ^{14}N Col/^{15}N *acinus-2 pinin-1* sample and 9 pairs are displayed. The top chromatograph represents ^{14}N light fragment ions, and the bottom represents the ^{15}N heavy fragment ions. The apex of the heavy peak is centered with the dashed line. The light and heavy peptide almost co-elute, with the heavy version elutes about 2-4 seconds earlier than the light version.

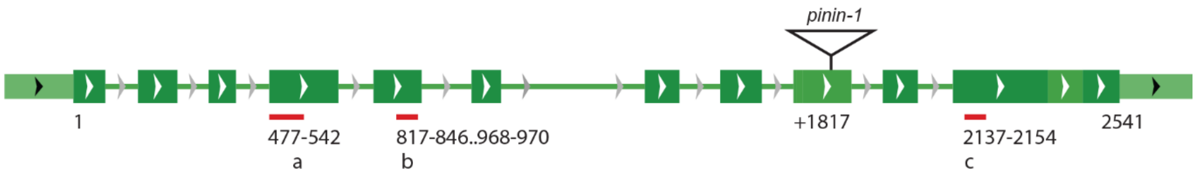

a $_{81}$VDGEDVSKDGEFPVDGNGTQVK$^{3+}{}_{102}$ Normalized ratio (*ap*/Col)=0.0%, 0.0%, 0.0%

b $_{166}$MLGNLLGTLEK$^{2+}{}_{176}$ Normalized ratio (*ap*/Col)=0.0%, 0.0%, 0.0%

c $_{290}$TFLEWK$^{2+}{}_{295}$ Normalized ratio (*ap*/Col)=0.0%, 0.0%, 0.0%

Supplemental Figure 4: Targeted quantification using PRM on PININ proteins show *pinin* mutant is a null allele. Two peptides and one peptide in regions before and after the T-DNA insertion were used for the targeted analysis. PRM data shows that both the N and C-termini of PININ were detectable in Col but not detectable in the *acinus-2 pinin* double mutant (Bi et al., 2021).

Supplemental Method | Method used for the second-batch sample preparation and data acquistion

The WT and *acinus-2 pinin-1* plants were grown on Hoagland medium containing ^{14}N or ^{15}N (1.34 g/L Hogland's No. 2 salt mixture without nitrogen, 6 g/L Phytoblend, and 1 g/L KNO_3 or 1 g/L $K^{15}NO_3$ (Cambridge Isotope Laboratories), pH 5.8) as described as (Bi et al., 2021). Proteins were extracted from two samples (one ^{14}N-labeled Col, one ^{15}N-labeled *acinus-2 pinin-1)* followed with protocols as described (Xu et al., 2017) with slight modification. Briefly, samples were first extracted individually using SDS sample buffer (0.1 M Tris·HCl, pH 8.0; 2% (wt/vol) SDS; 20 mM EGTA; 20 mM EDTA; 1.2% (vol/vol) Triton X-100; 2x protease inhibitor), then the protein concentration of each sample was measured by BCA assay kit (Thermo Fisher), subsequently followed by mixture to have 1:1 protein concentration mixture. Then Proteins were further extracted by cold phenol extraction. Proteins were digested with trypsin and the resulting peptides were de-salted using Sep-Pak waters C18 centrifuge columns. The peptides were analyzed on a Q-Exactive HF mass spectrometer (Thermo Fisher) equipped with an Easy LC 1200 UPLC liquid chromatography system (Thermo Fisher).

Data dependent Acquisition: Peptides were first trapped using trapping column Acclaim PepMap 100 (75 uM x 2cm, nanoViper 2Pk, C18, 3 µm, 100A), then separated using analytical column Acclaim PepMap RSLC (75um x25cm, nanoViper, C18, 2 µm, 100A) (Thermo Fisher). The flow rate was 300 nL/min, and a 120-min gradient was used. Peptides were eluted by a gradient from 3 to 28% solvent B (80% (v/v) acetonitrile/0.1% (v/v) formic acid) over 100 min and from 28 to 44% solvent B over 20 min, followed by a short wash at 90% solvent B. For DDA acquisition, the precursor scan was from mass-to-charge ratio (m/z) 375 to 1600 and top 20 most intense multiply charged precursors were selected for fragmentation. Peptides were fragmented with higher-energy collision dissociation (HCD) with normalized collision energy (NCE) 27. DDA data was analyzed as described in (Shrestha et al., 2021).

PRM Targeted quantification:
The data-dependent acquisition was used first to get the peptide information from multiple proteins with peptide mass/charge (m/z), retention time, and MS2 fragments. 42 paired peptides were picked that span the whole elution profile of 130 minutes. For targeted analysis, parallel reaction monitoring (PRM) acquisition using a 10-min window (±5 min) was scheduled with an orbitrap resolution at 60,000, AGC value 2e5, and a maximum fill time of 60 ms. The isolation window for each precursor was set at 2.0 m/z unit. PRM data was processed with a 5-p.p.m. window using skyline from ^{14}N- and ^{15}N-labeled samples.

REFERENCE

Bi, Y., Deng, Z., Ni, W., Shrestha, R., Savage, D., Hartwig, T., Patil, S., Hong, S.H., Zhang, Z., Oses-Prieto, J.A.*, et al.* (2021). Arabidopsis ACINUS is O-glycosylated and regulates transcription and alternative splicing of regulators of reproductive transitions. *Nat Commun* 12, 945. 10.1038/s41467-021-20929-7

Shrestha, R., Reyes, A.V., Baker, P.R., Wang, Z.Y., Chalkley, R.J., and Xu, S.-L. (2021). 15N metabolic labeling quantification workflow in Arabidopsis using Protein Prospector. *bioRxiv*. 10.1101/2021.11.30.470624

Xu, S.L., Chalkley, R.J., Maynard, J.C., Wang, W., Ni, W., Jiang, X., Shin, K., Cheng, L., Savage, D., Huhmer, A.F.*, et al.* (2017). Proteomic analysis reveals O-GlcNAc modification on proteins with key regulatory functions in Arabidopsis. *Proc Natl Acad Sci U S A* 114, E1536-E1543. 10.1073/pnas.1610452114
